# The effect of Punicalagin on tongue carcinoma cells viability and angiogenesis by regulating TGF-β signalling pathway: an in vitro study

**DOI:** 10.1038/s41598-026-61164-8

**Published:** 2026-07-10

**Authors:** Suzan Seif Allah Ibrahim, Shahenda Mahmoud Farid, Iman Fathy, Nashwa El-Khazragy, Doaa Adel-Khattab

**Affiliations:** 1 Oral Medicine, Periodontology and Oral Diagnosis, Faculty of Dentistry, Ain Shams and Nahda University, Cairo, Egypt; 2 Oral Medicine, Periodontology and Oral Diagnosis, Faculty of Dentistry, Ain Shams University, Cairo, Egypt; 3 Oral Biology Department, Faculty of Dentistry, Ain Shams University, Cairo, Egypt; 4 Clinical Pathology and Hematology and Ain Shams Medical Research Institute (MARSI), Faculty of Medicine, Ain Shams University Cairo, Cairo, Egypt

**Keywords:** Punicalagin, Oral cancer, TGF-β pathway, Anti-angiogenic, Cancer angiogenesis, Biochemistry, Cancer, Cell biology, Molecular biology, Oncology

## Abstract

Punicalagin (PUN) is a major bioactive ellagitannin derived from pomegranate and showed a potential anticancer property on several cell lines, no studies investigate its effect on tongue carcinoma cells. The present study aimed to evaluate the cytotoxic and antiangiogenic effects of PUN on human tongue carcinoma cell line. The viability of the tongue carcinoma cell line was assessed following treatment with PUN (250, 200, 150, 100, and 50 µmol) for 24 h using MTT assay. In addition, the calculated (IC 50) was used for apoptosis validation using Annexin-V/Propidium Iodide (PI) flow cytometry, angiogenesis investigation using endothelial tube formation assay, and transforming growth factor-β (TGF-β) expression on human tongue carcinoma cells after treatment with Punicalagin using quantitative real-time polymerase chain reaction (qRT-PCR). The vitality of tongue carcinoma cells treated with PUN was 61.1 ± 4.36% and the untreated cells were 98 ± 2%. Flow cytometry confirmed that treatment with 185 µmol/mL of PUN induced significant apoptosis, yielding 51.3% apoptotic cells, 2.3% necrotic cells, and 46.7% viable cells. Regarding tubules no. HPF Untreated cells had a statistically significant higher value (5.83 ± 1.94) than cells treated with PUN (3.17 ± 1.17) (*p* = 0.023). The junction no/HPF had a higher value in untreated cells (5.50 ± 2.43) than in cells treated with PUN (3.33 ± 1.37) yet the difference was not statistically significant (*p* = 0.119). The Tube length showed a statistical significance between untreated cells (5.28 ± 0.77) and cells treated with PUN (1.83 ± 0.26) (*p* = 0.004). The values for lobules no/HPF were higher in untreated cells (2.67 ± 0.52) than in cells treated with PUN (1.67 ± 0.82) yet the difference was not statistically significant (*p* = 0.053). The expression of TGF-β gene showed a significantly higher value in untreated cells (0.97 ± 0.03) than in cells treated with PUN (0.02 ± 0.01) (*p* = 0.004). These initial in vitro observations indicate an anticancer effect of PUN on tongue carcinoma cells through the induction of apoptosis and inhibition of cell viability and angio-mimetic tube formation, closely correlating with the downregulation of TGF-β gene expression. This provides a possible preliminary therapeutic direction to the study of tongue carcinoma.

## Introduction

Squamous cell carcinoma (SCC) is the most diagnosed malignant neoplasm in the oral cavity^1^, the number of cases of oral tongue squamous cell carcinoma (OTSCC) has increased in recent decades^2^. Early diagnosis of OTSCC leads to better treatment outcomes, but less than 40% of cases are diagnosed early. Despite advancements in treatment, the survival rate for advanced OTSCC is still low, with a five-year survival rate ranging from 30% to 50%^3,4^.

OTSCC is considered aggressive and difficult to treat because the tongue is enriched with vascular and lymphatic networks and therefore, more prone to progression, invasion and metastasis^5^. Surgery and chemotherapy are the most commonly used therapeutic methods for cancer and are successful in the treatment of many types of cancer but have many adverse effects^6^. Herbal medicines typically exhibit fewer adverse side effects and can enhance the therapeutic efficacy of conventional radiotherapy and chemotherapy when used in combination^7^.

Punicalagin (PUN) is the most bioactive polyphenols found in pomegranate fruits and is responsible for > 50% of the juice’s potent antioxidant activity, antimutagenic activity, antiproliferative and also protect DNA^8,9^. Punicalagin has anti-angiogenic properties by preventing the development of blood vessels in a variety of cancer cell lines including osteosarcoma, leukemia and prostate cancer cells^10,11^.

Transforming growth factor-β (TGF-β) functions as an indirect angiogenic factor by regulating the gene expression of VEGFs at the transcription stage promoting angiogenesis^12–14^. TGF-β is secreted in the tumour microenvironment and plays an important role in many cellular pathways, including proliferation, differentiation, migration and apoptosis^15^. In carcinogenesis TGF-β signalling disruptions are associated with poor prognosis due to the induction of epithelial-mesenchymal transition (EMT) through upregulation of matrix metalloprotease 9 (MMP-9) levels^16^. In transgenic mice, overexpression of TGF-β1 was reported to result in the hyperproliferation of cells at the head and neck epithelium and to enhance inflammation and angiogenesis^17,18^, it is suggested that TGF-β promoted cell proliferation by the formation of an extracellular microenvironment in favour of tumour formation, even at the early stage of carcinogenesis^19^. In transgenic mouse models, overexpression of TGF-β1 induces hyperproliferation within the head and neck epithelium while accelerating inflammation and angiogenesis. This suggests that TGF-β drives cell proliferation by establishing a supportive extracellular microenvironment that favors tumor development, even during early carcinogenesis.

To the best of our knowledge, this is the first study to investigate the anti-angiogenic effect of PUN on tongue carcinoma via regulation of TGF-β. Targeting the TGF-β pathway could be a new and effective way to improve the effectiveness of standard anticancer drugs against tongue carcinoma. This study hypothesizes that PUN can inhibit the formation of new blood vessels in tongue carcinoma by down-regulating TGF-β.

## Materials and methods

This in vitro study was conducted in the Faculty of Dentistry Ain Shams University and approved by the ethical committee with registration code and informed consent was done (FDASU-RecEM122007).

**Fig. 1:**
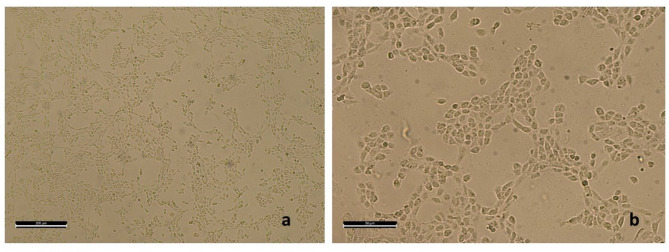
Photomicrograph of Tongue cancer cell culturing under inverted phase contrast microscope. (**a**) cells under magnification ×100, (**b**) Cells under magnification ×200.

The HNO-97 cells were purchased from ATCC (ATCC: American Type Culture Collection, Manassas, VA, USA). Cells were cultured in Dulbecco’s Modified Eagle Medium (DMEM) (Gibco, Thermosientific, Germany) containing 10% fetal bovine serum FBS (Gibco, Thermosientific, Germany) and 1% of penicillin G sodium (10.000 UI), streptomycin (10 mg) and amphotericin B (25 µg) (Gibco, Thermosientific, Germany) and were incubated at 37 °C a humidified atmosphere of 5% CO2, 95% air in an incubator (Thermo scientific- HERA cell VIOS 160i CO2 incubator with IR sensor). Photomicrographs of HNO-97 cells were captured to qualitatively evaluate baseline cancer cell morphology, cell adherence, and cellular density prior to experimental treatments utilizing a Leica DMi8 automated inverted fluorescence microscope as shown in Fig. 1. Punicalagin^®^ ≥98% (HPLC) (cat no: P0023-1 mg) (Sigma Aldrich – Germany) was obtained as a lyophilized powder and reconstituted according to the manufacturer’s instructions. PUN was mixed with 216.9 µL of methanol (Sigma Aldrich – Germany) to form a 500µL stock solution of PUN. A stock solution of 500 mM was prepared and aliquoted and stored at − 20 °C until use. The stock solution was diluted to different serial concentrations (250µmol, 200 µmol, 150 µmol, 100 µmol, 50 µmol) with PBS. The HNO97 cells were seeded in a 96-well culture plate. An average of 1 × 10^4^ HNO97 cells were seeded in 200 µL of DMEM containing 10% FBS and 1% of penicillin G sodium (10.000 UI), streptomycin (10 mg) and amphotericin B (25 µg). Culture plates were incubated at 37 °C in an atmosphere of 5% CO2 for 24 h to reach the 70% confluence. HNO-97 cells were treated with serial concentrations of PUN to evaluate its direct, dose-dependent effects on cancer cell viability treated with serial concentrations of PUN. While in the control group, the carrier solvent methanol was added to the cells and then incubated.

### Determination of cytotoxicity by MTT assay

The cytotoxic activity of PUN was examined via an MTT-dependent colorimetric assay^20^. The cells were treated with serial concentrations of PUN and later placed in incubation for around 24 h at 37 °C. After that, the MTT solution was mixed into all the wells. Then, the plates were placed and incubated around 4 h at 37 °C. As a result, the medium was detached and DMSO (100 ml) was mixed with the well in an attempt to mix the formazan crystals. Lastly, the absorbance was determined at the range of 570 nm by the use of a spectrophotometer (ELx 800; Bio-Tek Instruments Inc., Winooski, VT, USA)^21^.

The half maximal stimulatory concentration (IC50) was calculated using the Graph pad prism software 9. As a confirmatory test cell counting via trypan blue exclusion was performed following treatment with the (IC 50) concentration of PUN (185 µmol) using an automated cell counter (Thermo Scientific, Countess II automated cell counter) to estimate the total number of cells. Negative trypan blue-stained rounded cells indicated membrane integrity and cell viability, while positively stained cells indicated dead cells.

### Flow cytometric evaluation of apoptosis

HNO-97 cells were treated with the calculated I(C 50) dose (185 µmol/mL) of Punicalagin for 48 h. Cells were harvested, washed with PBS, and stained using Annexin-V FITC and Propidium Iodide (PI) for 15 min in the dark. The distribution of live, apoptotic, and necrotic cell populations was quantified using a flow cytometer.

### Tube formation assay

Following the baseline cell handling protocols, endothelial cells were prepared for the functional tube formation assay. The calculated (IC50) of PUN (185 µmol) was dissolved in the recommended basal medium. A 1 mL volume of each respective test or control medium was utilized to resuspend 1.5 × 10^5^ endothelial cells till they showed 70–90% confluency the medium was removed from the culture vessel and washed the cells twice with PBS. The washing solution was removed and 50 µL of accutase solution (Sigma Aldrich, Germany) was added per cm^2^ of vessel surface. The vessel was closed and incubated at 37 °C for 3–5 min and cells were examined under a microscope. The side of the vessel was gently tapped to accelerate cell detachment. When about 80% of the cells had detached, 100 µL of growth medium per cm^2^ of vessel surface was added and gently pipetted up and down to generate a single cell suspension then transferred to an appropriate centrifuge tube and rinsed the vessel surface again with 100 µL endothelial cell growth medium (Sigma Aldrich, Germany) per cm^2^ of vessel surface to collect remaining cells then cell number was counted by hemocytometer. 15 mL centrifuge tubes were prepared (one tube for negative control and another test tube treated with IC50 of PUN) and 1 × 10^5^ cells were of endothelial cells in each tube. Then the tubes were centrifuged at 300 xg for 3 min and each pellet was resuspended in 1 mL of the corresponding test or control medium.100 µL (= 1 × 10^4^ cells) of each single cell suspension were added per well on top of the gelled basement membrane extract (BME) (Thermo Fisher Scientific- US) without touching the surface of the gel and incubated. Cells were monitored using an inverted microscope. Several images were captured from each well using Labomed inverted microscope, USA. Images were captured with magnification power 40X, scale bar 100 μm. Further assessment was performed by analysing the number of tubules, number of junctions, tubule length, and number of lobules, the images were analysed using the Wimasis WimTube analysis tool (Wimasis GmbH Munich, Germany).

Total RNA was extracted from control and treated (24 h) HNO-97 cells using the RNeasy Mini Kit (Qiagen, Germany) and reverse transcribed with the Quantitect RT Kit (Qiagen, Germany). Amplification was conducted with the Quantitect primer assay [Hs_TGF-β] using Quantitect SYBR Green Master Mix (Qiagen, Germany) on a 5-plex Rotor-Gene PCR Analyzer (Qiagen, Germany). Relative fold changes were calculated via the 2^∆∆Ct^ method using GAPDH as the internal normalization reference control. Primer specificity was verified through raw dissociation curve mapping and derivative melting peak profiling.

### Statistical analysis

Data management and statistical analysis were performed using the Statistical Package for Social Sciences (SPSS) version 18. Numerical data were summarized using mean, standard deviation and confidence intervals. Data were explored for normality by checking the data distribution and using Kolmogorov-Smirnov and Shapiro-Wilk tests. Comparisons between treated and untreated HNO97 cells concerning normally distributed numeric variables were performed by independent t-test, while the MTT assay of different treatment concentrations was performed using ANOVA test and Tukey’s post hoc test. All p-values are two-sided. P-values ≤ 0.05 were considered significant.

## Results

### Cytotoxic effects of PUN on human tongue cancer cell line

The antiproliferative activity of PUN against HNO-97 cells was quantitatively evaluated via the MTT colorimetric assay. PUN exposure induced a dose-dependent suppression of HNO-97 cell proliferation compared to untreated control groups, confirming a distinct inverse correlation between extract concentration and metabolic cell viability. Quantitative cell survival distributions across the tested cohorts revealed that the highest viability baseline was maintained in untreated cells (99.64%), which progressively declined in groups treated with 50 μm (91.28%), 100 μm (76.03%), 150 μm (60.44%), and 200 μm (45.95%) of PUN. The lowest cellular survival threshold was recorded in the cohort exposed to the highest concentration of 250 μm PUN (38.74%), systematically validating that cellular viability diminishes sequentially with escalating treatment doses (Table 1).

**Table 1:** MTT assay results for HNO-97 cells treated with different concentrations of Punicalagin using optical density 570 nm after 48 h duration.

	NC [DMEM]	50 µmol/mL	100 µmol/mL	150 µmol/mL	200 µmol/mL	250 µmol/Ml
Mean	1.30^a^	1.19^b^	.99^c^	.79^d^	.60^e^	.50^f^
SD	0.03	0.06	0.06	0.04	0.05	0.01
95% C.I.	1.27 to 1.32	1.14 to 1.23	0.95 to 1.03	0.76 to.82	0.56 to.64	0.50 to.51
P value	0.000*					
% of viability	99.64%	91.28%	76.03%	60.44%	45.95%	38.74%

*; significant (*p* ≤ 0.05) ns; non-significant (*p* > 0.05).

## Half-maximal cytotoxic concentration (IC50) and apoptosis profiling of PUN

**Fig. 2:**
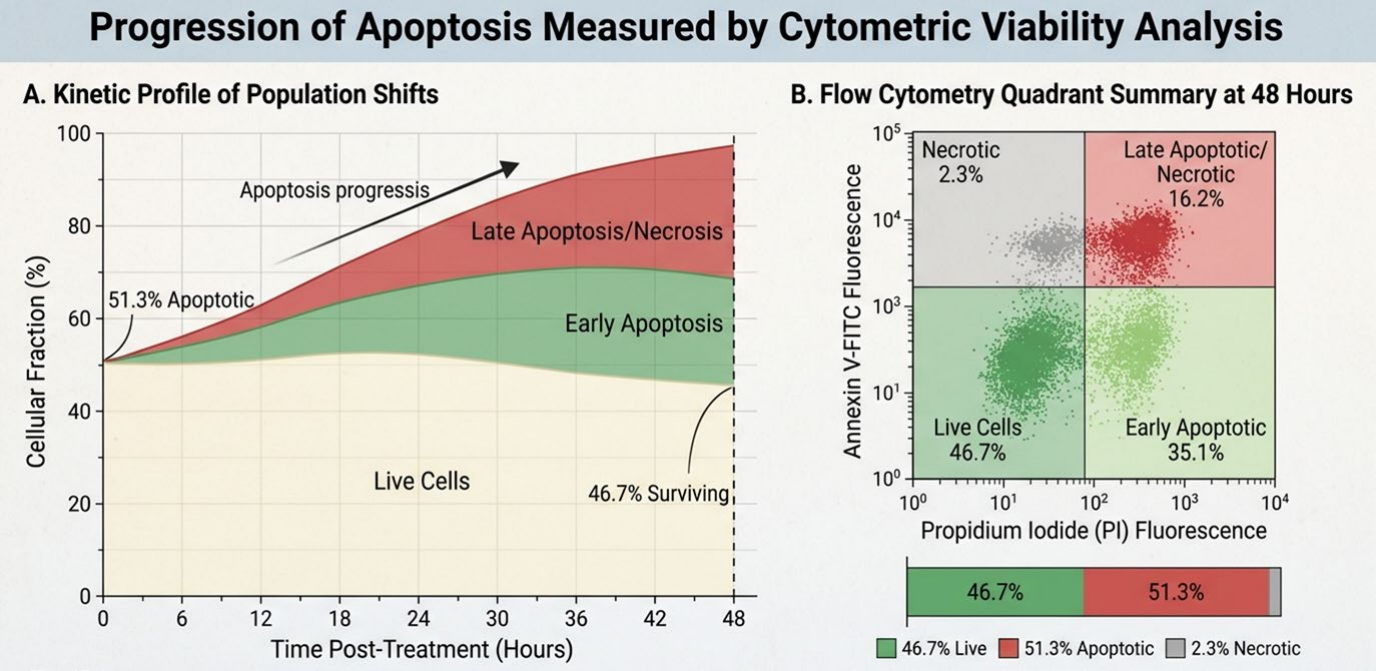
Flow cytometric characterization of cellular apoptosis induction. (**A**) Kinetic profile of cellular population shifts tracking the time-dependent progression of HNO-97 tongue carcinoma cells across a 48-hour post-treatment window. Over the incubation timeline, the viable cellular fraction steadily declines to a final baseline of 46.7% surviving cells, accompanied by a reciprocal elevation in early and late apoptotic populations reaching a combined total of 51.3% apoptotic cells. (**B**) Representative Annexin V-FITC / Propidium Iodide (PI) dual-staining quadrant fluorescence dot plot evaluated at 48 h post-treatment. Analytical quadrant gating quantifies the definitive cell distribution metrics: Live Cells localized in the bottom-left quadrant (46.7%), Early Apoptotic cells in the bottom-right quadrant (35.1%), Late Apoptotic/Secondary Necrotic cells in the top-right quadrant (16.2%), and Primary Necrotic debris in the top-left quadrant (2.3%). The bottom summary tracker confirms a total apoptotic transformation of 51.3% against a minimized necrotic background index.

The half-maximal inhibitory concentration (IC50) of PUN against the HNO-97 line was determined to be 185 μm, with a 95% confidence interval (CI) spanning from 165.4 to 216.5 μm (Fig. 1). Annexin-V/PI dual-staining flow cytometry was executed to characterize the specific mode of cell death driving this cytotoxic response. Quantitative quadrant analysis revealed a distinct pro-apoptotic response, where exposure to the 185 μm threshold significantly shifted the cell population toward active programmed death, yielding 51.3% apoptotic cells and a controlled necrotic fraction of only 2.3%, while the surviving viable cell population was reduced to 46.7% (Table 2; Fig. 2).

**Table 2:** Intergroup comparison, mean and standard deviation (SD) values for cell viability% for different groups.

Untreated cells	Cells treated with Punicalagin	*p*-value
98.00 ± 2.00	61.00 ± 4.36	0.100ns

*; significant (*p* ≤ 0.05) ns; non-significant (*p* > 0.05).

## PUN attenuates angiogenesis of tongue carcinoma cells

**Fig. 3:**
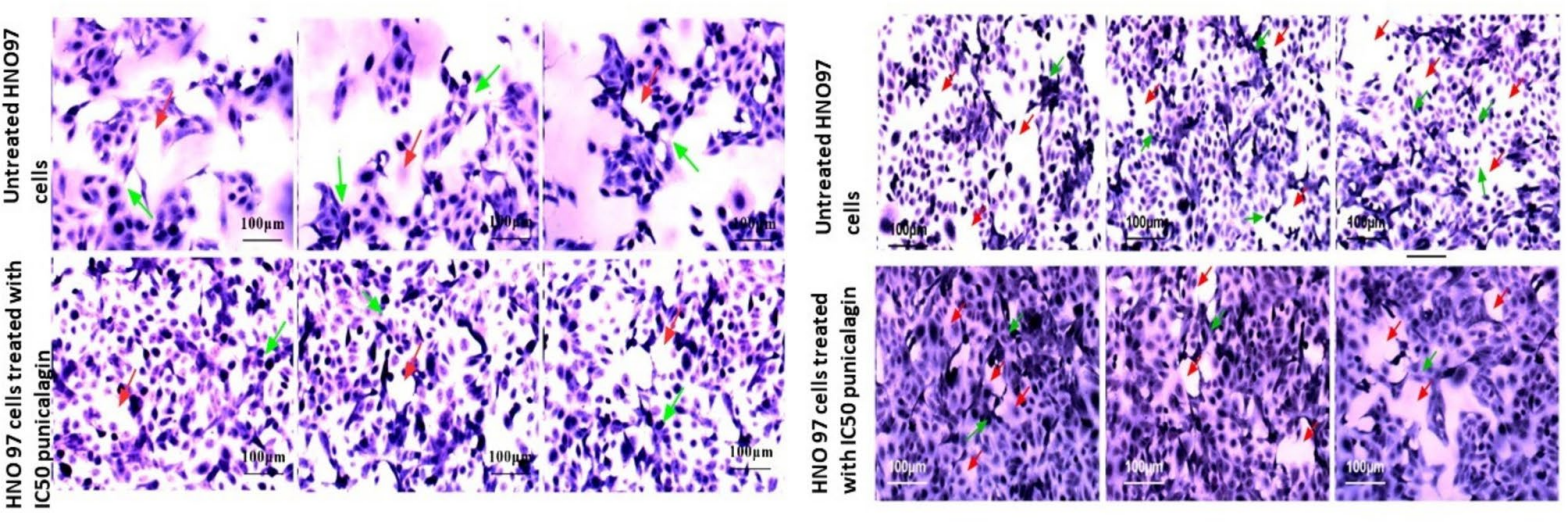
In vitro angiogenesis assay Wimasis WimTube generated image of HNO97 cells grown on extracellular matrix in 6-well culture plate in 6 sample groups of treated and untreated HNO-97 cells in triplicates. The untreated HNO-97 cells showed characteristics of tube formation in form of lobules (red arrow), tubular junctions (green arrow), however, the HNO97 cells that were treated with 185.0 µmol/mL of PUN showed few numbers of tubules with short tubular length, which reflect inhibition of new blood vessel formation induced by PUN. Images were taken using Labomed inverted microscope, USA. Images are captured with magnification power 40X, scale bar 100 μm.

An in vitro endothelial tube formation assay was conducted to assess the potential anti-angiogenic properties of PUN. Untreated control cells displayed an advanced, complex endothelial network architecture characterized by the organized configuration of closed loops, capillary-like lobules, and distinct tubular junctions. Conversely, HNO-97 cells subjected to the calculated IC50 threshold (185 μm) of Punicalagin exhibited severe network disruption, forming sparse tubular sprouts with severely truncated tube lengths, indicating a potent inhibition of angio-mimetic capabilities (Fig. 3).

Quantitative structural profiling utilizing automated Wimasis network tracing tools validated these significant variations:

- Number of Tubules/HPF: The untreated control group exhibited a significantly higher structural count (5.83 +/- 1.94) compared to the PUN-treated cohort (3.17 +/- 1.17; *p* = 0.023).
- Total Tube Length: Network length metrics demonstrated a highly significant suppression, dropping from an untreated baseline of 5.28 +/- 0.77 mm/HPF down to 1.83 +/- 0.26 mm/HPF in the treated environment (*p* = 0.004).
- Number of Junctions/HPF: Untreated samples displayed higher connectivity counts (5.50 +/- 2.43) relative to the PUN-exposed samples (3.33 +/- 1.37), although this architectural variation did not reach strict statistical significance (*p* = 0.119).
- Number of Lobules/HPF: Closed loop counts were higher in the control microenvironment (2.67 +/- 0.52) compared to the treated samples (1.67 +/- 0.82), bordering the threshold of statistical significance (*p* = 0.053; Table 3).

**Table 3:** Intergroup comparison, mean and standard deviation (SD) values for tubules no/HPF for different groups.

Untreated cells	Cells treated with Punicalagin	*p*-value
**Tubules no/HPF (Mean ± SD)**		
5.83 ± 1.94	3.17 ± 1.17	0.023*
**Junction no/HPF (Mean ± SD)**		
5.50 ± 2.43	3.33 ± 1.37	0.119ns
**Tube length (mm) (Mean ± SD)**		
5.28 ± 0.77	1.83 ± 0.26	0.004*
**Lobules no/HPF (Mean ± SD)**		
2.67 ± 0.52	1.67 ± 0.82	0.053ns

*; significant (*p* ≤ 0.05) ns; non-significant (*p* > 0.05).

### PUN downregulates the TGF-beta signaling pathway in HNO-97 cells

**Fig. 4:**
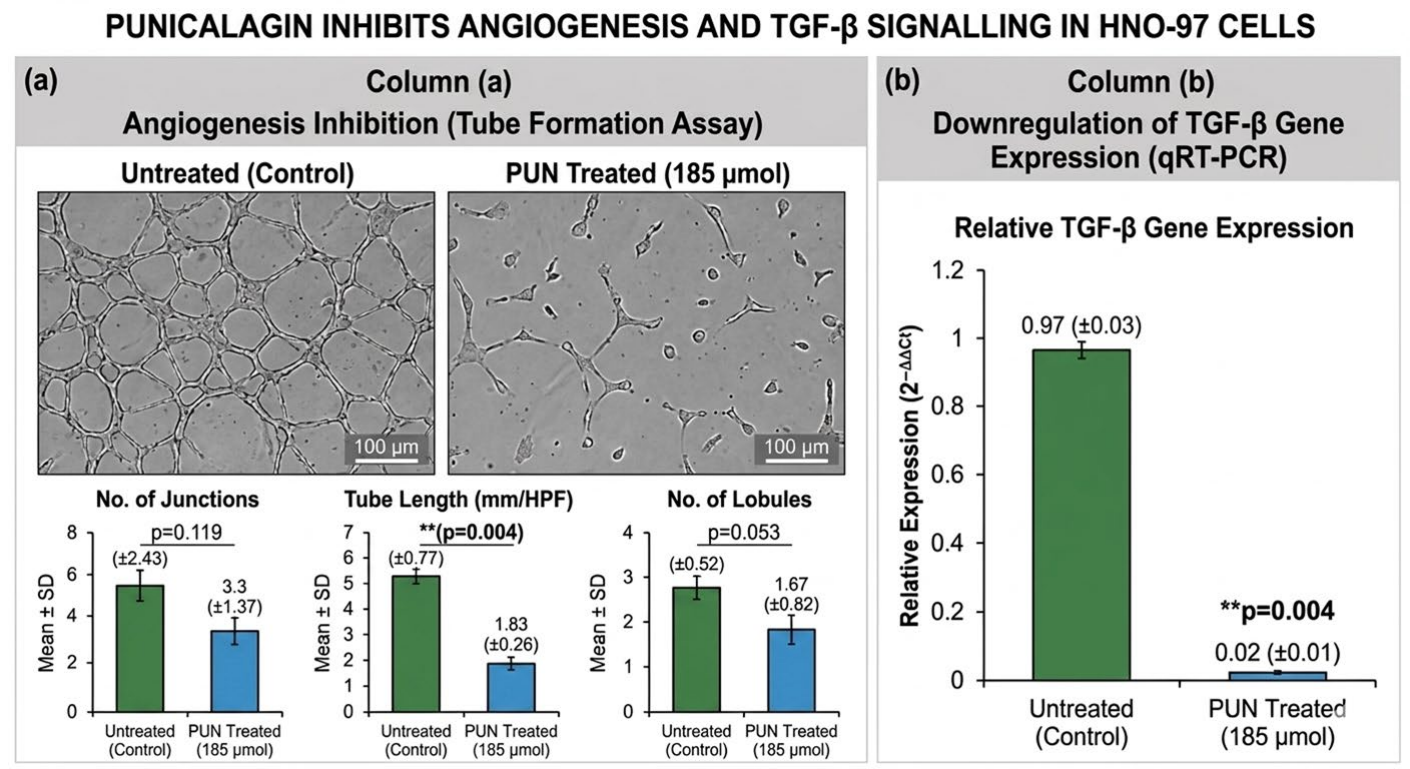
Punicalagin suppresses angio-mimetic networks and downregulates the TGF-β transcript profile.(a) Functional in vitro angiogenesis evaluation via endothelial tube formation assay. Top panel captures bright-field inverted phase-contrast micrographs showing baseline complex loops and networks in the Untreated (Control) HNO-97 cell environment versus fragmented structural branches in the PUN-Treated (185 µmol) group (scale bars = 100 μm). Bottom panel bar charts represent quantitative Wimasis metrics comparing the Mean ± SD of No. of Junctions (*p* = 0.119), Tube Length (**p = 0.004), and No. of Lobules (*p* = 0.053) per high-power field (HPF).(b) Quantitative real-time PCR expression profiling of the target signaling cascade. The bar chart depicts relative fold-expression changes (2^-ΔΔCt) for the TGF-β transcript, highlighting a highly significant transcriptional downregulation from a baseline of 0.97 ± 0.03 down to 0.02 ± 0.01 (**p = 0.004) post-treatment. Data represent Mean ± SD of independent biological experiments executed in triplicate.

**Fig. 5:**
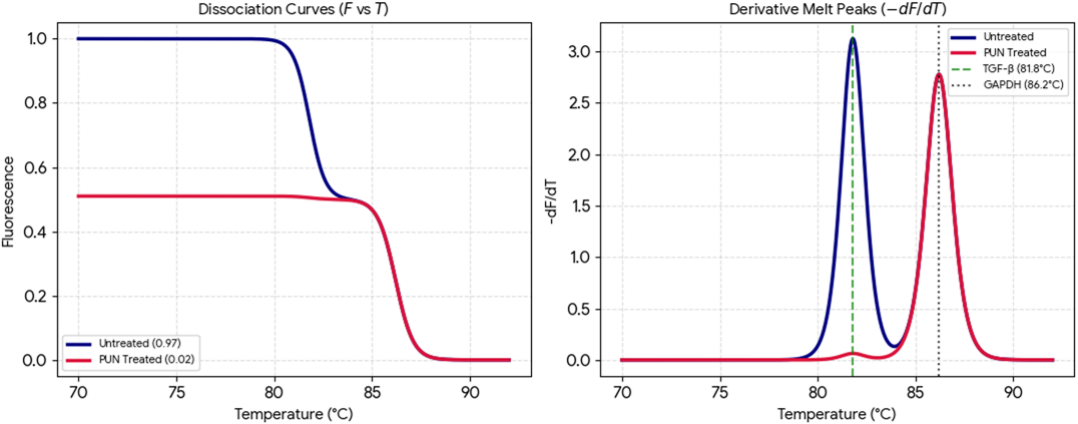
Real-time quantitative PCR melt curve and product dissociation profiles. Left Panel: Raw dissociation curve mapping tracking cellular fluorescence intensity (F) against Temperature (°C) for the Untreated (0.97 relative baseline) and PUN Treated (0.02 relative baseline) HNO-97 cell lines. Right Panel: Quantitative derivative melt peak profile (-dF/dT versus Temperature) demonstrating strict gene-specific sequence targeting. The presence of single, distinct clean amplification curves identifies the specific target melting temperature (Tm) of the TGF-β gene at 81.8 °C (green dashed line) and the internal housekeeper GAPDH reference control gene at 86.2 °C (black dotted line), confirming the complete absence of secondary non-specific amplicons or primer-dimer artifacts.

Quantitative real-time PCR (qRT-PCR) analysis was conducted to clarify the transcriptional mechanisms associated with the observed phenotypic changes. Relative mRNA fold-expression transitions, normalized against the internal housekeeper reference gene (GAPDH), demonstrated that PUN treatment induced a dramatic transcriptional silencing of the target pathway. The relative expression abundance of the TGF-beta transcript in the untreated control group was recorded at a baseline value of 0.97 +/- 0.03, which was markedly ablated down to 0.02 +/- 0.01 following a 24-hour exposure to Punicalagin, proving a highly statistically significant transcriptional downregulation (*p* = 0.004; Table 4; Fig. 4). Primer specificity was verified via post-amplification dissociation curves, capturing single symmetrical derivative melt peaks for the target TGF-beta gene at 81.8 degrees C and the reference GAPDH control at 86.2 degrees C with no evidence of non-specific artifacts (Fig. 5).

**Table 4:** Intergroup comparison, mean and standard deviation (SD) values for fold change of TGF-β gene expression (PCR) for different groups.

TGF-β gene expression (PCR) (Mean ± SD)		*p*-value
Untreated cells	Cells treated with Punicalagin	
0.97 ± 0.03	0.02 ± 0.01	0.004*

*; significant (*p* ≤ 0.05) ns; non-significant (*p* > 0.05).

## Discussion

Natural polyphenol’s anticancer efficacy has been attributed largely to their powerful anti-inflammatory and antioxidant activities, as well as their ability to control molecular receptors and signalling pathways associated with cellular growth, proliferation, differentiation, invasion, angiogenesis, and immune responses due to polyphenols which consist mainly of hydrolyzed tannins^22^. There is growing research regarding the components of pomegranate (Punica granatum) in terms of healthy nutrition and medicine due to the higher antioxidant activity compared to vitamin E, β-carotene and ascorbic acid^23,24^.

PUN has been reported to inhibit proliferation and angiogenesis in osteosarcoma cells through suppression of NF‑κB signalling and also induced cell apoptosis^10^. Another study reported that pomegranate juice (PJ) and pomegranate tannin extract, which are both rich in PUN, successfully inhibited the proliferation of human oral cancer cell lines such as KB and CAL27^9^. Consequently, the present study aimed to investigate the possible antiangiogenic, cytotoxic, and apoptosis-inducing effects of PUN on the human tongue carcinoma cell line (HNO-97) and evaluate the effect on tube formation and regulation of the TGF-β gene in PUN-treated cells in comparison to untreated control cells.

The results of this study showed that PUN significantly inhibited proliferation of tongue carcinoma cells (HNO-97) in a concentration-dependent manner, the viability percentage means decreased from 91.28% to 76.03% to 60.44% to 45.95% to 38.74% progressively as the concentration of PUN increased from 50 μm to 100 μm to 150 μm to 200 μm to 250 μm consequently. These results are compared to normal cells that showed 99.64% viability. These findings indicated that increasing the concentration of PUN is highly toxic to viable cultured cells, an observation in accordance with that reported in the study by Zhang et al. who reported that PUN has clear cytotoxicity to human cervical cancer (ME-180 cells) in a concentration dependant mode (10 to 100 μm). In Zhang’s study^24^ the PUN caused around 80% reduction in cell viability compared to control cells in a dose-dependent manner. Another study on lung cancer cell lines which used lower concentrations of PUN than this study showed a higher percentage of decreased viability of cancer cells^25^. This conflict in Punicalagin concentrations may be due to the different responses of different cell types to treatment, besides, tongue cancer cells are more resistant to chemotherapy than other cancer cell types^26^.

In the present study, the anti-angiogenesis ability of PUN was assessed by using tube formation assay in vitro. This assay was used because it is rapid, reproducible, and quantitative. Moreover, number of tubules, number of junctions, tubule length and number of lobules can be analysed^21^. The results showed that PUN significantly decreased the number of tubules and tube length of blood vessels compared to untreated cells. However, the decrease in number of junctions and number of lobules were not statistically significant. These findings approved that PUN acts as an antiangiogenic drug in tongue carcinoma cells (HNO-97), this can be explained by that PUN inhibits vascular endothelial growth factor (VEGF) that is the main pro-angiogenic factor^27^. A previous study on the inhibitory effect of PUN on angiogenesis of human umbilical vein endothelium cells (HUVEC) concluded that pretreatment of cells with PUN inhibited the formation of blood vessel-like structures and also inhibited proliferation and migration of endothelial cells^28^. Another study on the effects of punica granatum juice (PGJ) in multiple myeloma showed that pomegranate juice in the presence or not of VEGF-A inhibited the formation of enclosed spaces between endothelium in blood vessels in Matrigel, it also affected the length of tubes and number of interconnections between tubes^29^. Another study on black pomegranate peel extract (PPE) on melanoma cells showed that PPE significantly reduced the size of the length and the number of junctions of the tubes relative to the negative control group^30^.

Since the colorimetric cell proliferation assay demonstrated only overall alterations in viability metrics, applying specialized validations to evaluate the precise path of death became critical. Our subsequent flow cytometry profiling using Annexin-V and PI tracking revealed a highly significant surge in apoptotic cell configurations up to 51.3% following treatment with the 185 μm dose. This explicitly illustrates that PUN-mediated suppression is orchestrated through apoptotic cell death mechanisms rather than passive cellular breaking, a finding that lines up closely with prior observations on human hepatoma configurations (HepG2) where matching concentrations induced ROS-driven mitochondrial apoptosis checkpoints.

To the best of our knowledge, this is the first study focusing on the role of TGF-β gene in the pathogenesis of tongue carcinoma. In this study, the expression of TGF-β in tongue carcinoma cells was confirmed, there was a statistically significant difference between HNO-97 cells treated group and untreated group. The quantification of TGF-β gene expression levels revealed a significantly lower fold change in the treated HNO-97 cell line group in comparison to the untreated group this suggests PUN caused down-regulation of TGF-β. These findings indicate that TGF-β may play an important role in the development of tongue carcinoma and could be regarded as a prognostic factor and suggest the importance of target gene functions in the process of tumor gene-targeted therapy. These results were in accordance with a study that showed that knockout of TGFBI by CRISPR/CAS-9 inhibited cell proliferation and clone formation, and enhanced cell migration and invasion in vitro on OSCC cell line and TGF-βI expression was higher in OSCC than in normal tissue and the survival rate of HNSCC patients with high TGF-βI expression was lower than that of patients with low expression therefore is significantly related to poor prognosis^18^. The present study showed that PUN had a cytotoxic and an antiangiogenic effect on the human tongue squamous cell carcinoma cell line (HNO-97) via regulation of TGF-β signalling pathway.

The primary limitation of this work is its strictly in vitro design, meaning complex systemic multi-organ microenvironments and systemic immune feedback networks were not evaluated. Furthermore, direct tracking of downstream protein signaling elements (such as Western blot or phospho-SMAD analysis) was not performed during this phase. Similarly, parallel pro-angiogenic cascades like VEGF signaling were not directly measured. The Clinical Translational Value & Future Directions, while these cell-line expression results show that Punicalagin exerts notable inhibitory checks on oral cancer cell behaviors, these findings cannot be directly translated to clinical outcomes without animal models. Future work will focus on expanding testing into in vivo settings, validating protein-level pathway checkpoints, tracking upstream Caspase activation profiles, and clarifying parallel VEGF network actions.

## Conclusion

This study concluded that PUN has a cytotoxic, pro-apoptotic, and antiangiogenic effect on human tongue carcinoma cell line (HNO-97) in vitro through induction of programmed apoptotic death, suppression of blood vessel formation profiles, and downregulation of the TGF-β gene pathway. Further extensive validation checks via complex in vivo animal modeling and protein signaling assays are required to firmly confirm and expand upon the precise translation of PUN therapeutic avenues.

## Data Availability

All data generated or analyzed during this study are included in this article. The datasets used and/or analyzed in the study are available from the corresponding author on reasonable request.
